# Pachymeningitis With Dural Vessel Dilatation in MOG Antibody–Associated Disease: A Case Report

**DOI:** 10.1155/crnm/5686302

**Published:** 2025-11-11

**Authors:** Yunchen Huang, Yafei Song, Ding Liu, Yin Liu

**Affiliations:** ^1^Department of Radiology, The Third Xiangya Hospital, Central South University, Changsha, China; ^2^Department of Neurology, The Third Xiangya Hospital, Central South University, Changsha, China

**Keywords:** dural, meningitis, myelin oligodendrocyte glycoprotein, seizure

## Abstract

**Background:**

Myelin oligodendrocyte glycoprotein (MOG) antibody–associated disease (MOGAD) is a novel inflammatory demyelinating disorder marked by heterogenous clinical and radiological manifestations. Pachymeningitis is a rare manifestation.

**Case Presentation:**

An 18-year-old male was hospitalized with fever, dizziness, altered consciousness, and seizure attacks. Serum testing was positive for MOG antibodies. Diffuse pachymeningitis with prominent dural vessel dilation was observed prior to treatment, which markedly improved after hormone therapy.

**Conclusion:**

MOGAD pachymeningitis with dural vessel dilatation broadens the imaging spectrum of MOGAD.

## 1. Introduction

Myelin oligodendrocyte glycoprotein (MOG) antibody–associated disease (MOGAD) is a recently recognized inflammatory demyelinating disease mediated by antibodies against MOG [1]. Typical clinical manifestations include optic neuritis, myelitis, acute disseminated encephalomyelitis, brainstem encephalitis, cortical encephalitis, and aseptic meningitis [1–3]. Radiological features of MOGAD are similarly diverse, reflecting the clinical phenotype [1]. Pachymeningitis represents a rare imaging manifestation of MOGAD, and this report describes a case of MOGAD with pachymeningitis and prominent dural vessel dilatation.

## 2. Clinical Data

**Figure 1:**
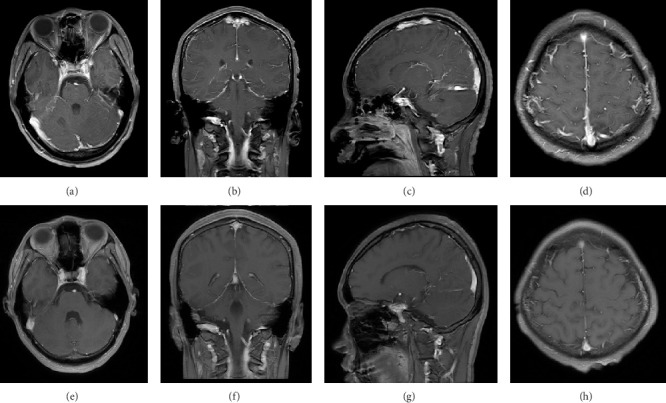
Postcontrast T1-weighted MR of the patient before (a–d) treatment showed diffuse and irregular pachymeningeal enhancement, which attenuated obviously after (e–h) treatment. Multiple dilated dural vessels can be seen between the cranium and brain surface (d), which were resolved after treatment (h).

An 18-year-old male patient was admitted to the hospital with a 4-day history of fever and dizziness, followed by 2 days of altered consciousness and seizure attacks. The patient was sedated upon admission, with equal and round pupils that were reactive to light. His neck was supple. A lumbar puncture was performed on the day of admission. The cerebrospinal fluid (CSF) pressure was found to be 185 mmH_2_O, with CSF analysis revealing total protein of 1632 mg/L and glucose of 5.19 mmol/L. Oligoclonal bands were negative. CSF cytology indicated a total white blood cell count of 110 × 10^6^/L (67% lymphocytes and 11% neutrophils). Infection work-up revealed IgG positivity for Epstein–Barr virus, *Mycoplasma pneumoniae*, and *Chlamydophila pneumoniae*, while both blood and CSF bacterial cultures were negative. Serum autoantibodies, including ANCA, ANA, rheumatoid factor, anti-SSA, and anti-SSB, were negative. An MRI of the head performed on the second day of admission revealed diffuse linear dural thickening and enhancement with prominent dural vessel dilatation; however, no other parenchymal lesion was identified in the brain (Figures 1(a), 1(b), and 1(c)). The initial diagnosis was probable viral meningoencephalitis with secondary epilepsy. The patient was treated with acyclovir for antiviral therapy, along with valproic acid and levetiracetam for seizure control. However, live cell–based assays subsequently detected positive serum MOG antibodies at a titer of 1:100, while CSF MOG antibodies were negative. An additional serum IgG4 test was not found to be significant.

Following confirmation of the diagnosis of MOGAD, the patient was treated with high-dose intravenous methylprednisolone, 1,000 mg for the initial three days, followed by half the previous dose every 3 days, transitioning to oral prednisolone at 60 mg/day. On the fifth day of intravenous methylprednisolone therapy, the patient regained consciousness and remained seizure-free. A follow-up lumbar puncture performed after 1 week of hormone treatment revealed a CSF pressure of 150 mmH_2_O and a total protein of 351 mg/L. CSF cytology was negative, with a total white blood cell count of 1 × 10^6^/L (no neutrophils). A follow-up contrast-enhanced brain MRI on the 12^th^ day of hormone treatment demonstrated complete resolution of dural thickening, enhancement, and dural vessel dilatation (Figures 1(d), 1(e), and 1(f)). No spinal MRI was performed, as the patient exhibited no spinal or optic symptoms.

## 3. Discussion

According to the proposed diagnosis criteria of MOGAD, this patient exhibited a typical pattern of meningitis, as evidenced by infection-like symptoms, CSF findings, and a positive serum MOG antibody at the titer of 1:100. However, this case demonstrated pachymeningeal involvement, which was different from the leptomeningeal pattern primarily reported in previous studies on the aseptic meningitis type of MOGAD [2].

Based on anatomic location and embryological origin, meningitis of the central nervous system can be classified into two patterns, pachymeningitis (involving the dura and dura-arachnoid interface) and leptomeningitis (pia-subarachnoid) [4]. Leptomeningitis typically presents with signs of meningeal irritation, whereas pachymeningitis is generally associated with headache, cranial nerve symptoms, seizures, and ataxia. Notably, oligodendrocytes are not normally present in the dura mater, which may explain the rarity of pachymeningeal involvement in MOGAD. However, cases of heterotopic neuroglial tissues have been reported [5].

Similar to the cases reported by Ueno et al. [6] and Khaladkar et al. [7], pachymeningitis was the only radiological finding in this case. However, the diffuse pachymeningeal enhancement pattern and infectious features observed here were not described in those reports. Consistent with the findings in Ueno et al. [6], the pachymeningeal enhancement resolved completely following high-dose intravenous methylprednisolone. However, the MOGAD patient with bilateral Sturge–Weber syndrome reported by Michishita et al. [8] showed bilateral diffuse pachymeningeal enhancement after treatment, while the concurrent right leptomeningeal enhancement and a focal lesion of the right cerebellar hemisphere resolved completely. The possibility that the bilateral dural enhancement in that case was influenced by the Sturge–Weber syndrome cannot be excluded. Moreover, prominent dural vessel dilatation was observed in the present case, which was not previously reported in MOGAD.

The mechanism underlying pachymeningeal enhancement, particularly the diffuse type, remains poorly understood. Intracranial volume compensatory changes seen in intracranial hypotension, as well as increased vascularity or angiogenesis secondary to meningeal pathology or irritation, may contribute to this phenomenon [9]. It has also been suggested that the diffuse pachymeningeal enhancement may be related to periosteal pathology [9]. Recent studies indicate that the vessels between the cranial bone and dura may be important passages for neuroimmune surveillance [10]. In this case, heterotopic neuroglial tissues within dura mater may be responsible for the pachymeningeal enhancement, while the dilated dural vessels as well as the pachymeningeal enhancement may reflect communication between the cranial bone marrow and meninges, suggesting a periosteal involvement in MOGAD meningitis.

Furthermore, the patient underwent MRI examination following the lumbar puncture procedure, which has been associated with diffuse leptomeningeal enhancement due to cerebral hypotension after lumbar puncture [11]. However, the patient did not report any symptoms suggestive of cerebral hypotension.

In summary, MOGAD pachymeningitis, with or without dural vessel dilatation, broadens the imaging spectrum of MOGAD. The dural enhancement pattern and its pathophysiological mechanism in MOGAD warrant further investigation.

## Data Availability

The data that support the findings of this study are available on request from the corresponding author. The data are not publicly available due to privacy or ethical restrictions.

## Nomenclature

ANA: Antinuclear antibody
ANCA: Antineutrophil cytoplasmic antibody
Anti-SSA: Anti-Sjögren's syndrome Type A antibody
Anti-SSB: Anti-Sjögren's syndrome Type B antibody
CSF: Cerebrospinal fluid
IgG: Immunoglobulin G
MOG: Myelin oligodendrocyte glycoprotein
MOGAD: Myelin oligodendrocyte glycoprotein antibody–associated disease
MRI: Magnetic resonance imaging
